# Supplementary material for: Personalized pathology test for Cardio-vascular disease: Approximate Bayesian computation with discriminative summary statistics learning
Source: PLoS Comput Biol. 2022 Mar 10;18(3):e1009910. doi: 10.1371/journal.pcbi.1009910 (PMC8939803; doi:10.1371/journal.pcbi.1009910)
Supplement: S1 Appendix — Details on Semi Automatic Summary Statistics Learning and Summary Statistics Learning by minimizing triplet loss. (PDF) [file pcbi.1009910.s001.pdf]

S1 Appendix: Additional mathematical details Personalized pathological test for Cardio-vascular disease: Approximate Bayesian computation with discriminativesummary statistics learning

Ritabrata Dutta[1]*, Karim Zouaoui Boudjeltia[2], Christos Kotsalos[3], Alexandre Rousseau[2], Daniel Ribeiro de Sousa[2], Jean-Marc Desmet[4], Alain Van Meerhaeghe[5], Antonietta Mira[6,7], Bastien Chopard[3],

[1] *Department of Statistics, Warwick University, Coventry, UK*

[2] *Laboratory of Experimental Medicine (ULB 222), Medicine Faculty, Université Libre de Bruxelles, ISPPC CHU de Charleroi, Charleroi, Belgium*

[3] *University of Geneva, Geneva, Switzerland*

[4] *Nephrology Department, ISPPC CHU de Charleroi, Charleroi, Belgium*

[5] *Pneumology Department, ISPPC CHU de Charleroi, Charleroi, Belgium*

[6] *Università della svizzera italiana, Lugano, Switzerland*

[7] *University of Insubria, Varese, Italy*

1 Details on Summary Statistics Learning

1.1 Semi Automatic Summary Statistics Learning

In semiautomatic summary statistics learning (SASL) schemes [1, 2], the parameter values are regressed using some function of the corresponding simulation outputs. Namely, you assume the following model:

$$\theta = \mathbb{E}(\theta|x) + \epsilon = f(x) + \epsilon, \tag{1}$$

where ϵ is a 0-mean noise and $f(x)$ is a function of data. The authors of [2] parametrize $f(\cdot)$ by using a Neural Network. This regression approach was first introduced in [1] with a linearity assumpton on f, reducing it to a simple linear regression. We focus here on the neural network formulation as this was shown to outperform the linear regression by [2].

In practice, we first simulate a 'pilot' set of n datasets $\{x_1, \ldots, x_n\}$ from n parameters $\{\theta_1, \ldots, \theta_n\}$ correspondingly and then fit the statistical model given by Eq. (1) to the simulated data. Then we consider $s(\cdot) = f_\beta(\cdot)$ as the summary statistics and Euclidean distance on this summary statistics space to define the distance for the ABC inference algorithm. Following [2], here we use a neural network $g_w(\cdot)$ with weights w to parametrize the function $f(\cdot)$, and that was trained by stochastic gradient descent using the loss corresponding to the regression in Eq.(1):

$$\frac{1}{N}\sum_{i=1}^{N} ||f_\beta(x_i) - \theta_i||_2^2. \tag{2}$$

In Theorem 3 of [1], the authors provide a rationale for the above procedure; namely, they show that, by using $s(x^0) = \mathbb{E}(\theta|x^0)$ as summary statistics, the posterior mean of the ABC approximate posterior is the best possible estimator of the true parameter value with respect to the quadratic error loss. Of course, the

*Corresponding author: Ritabrata.Dutta@warwick.ac.uk.

posterior mean with respect to the true posterior $\mathbb{E}(\theta|x^0)$ is not available, and hence the regression approach was proposed.

1.2 Triplet Loss Summary Statistics Learning

The summary statistics learning approach minimizing triplet loss (TLSL) was first introduced in [3], which considers the assumption that the geometry induced in data space by the Euclidean distance on the learned summary statistics ($s(x) = g_w(x)$ where $g_w(\cdot)$ is a neural network with weights w) should be similar to the geometry in the corresponding parameter space induced by Euclidean distance (d_E). After simulating a set of n datasets $\{x_1, \ldots, x_n\}$ from n parameters $\{\theta_1, \ldots, \theta_n\}$ correspondingly, to learn the weights of the neural networks, here we consider the triplet [4] loss. The triplet loss works on three samples at a time: an anchor, a positive, that is deemed similar to the anchor, and a negative, that is on the contrary dissimilar. Essentially, the loss pushes the network to find an embedding such that the distance between the anchor and the negative is larger than the one between the anchor and the positive plus a margin, that is defined a priori. By denoting $(x_a^{(i)}, x_p^{(i)}, x_n^{(i)})$ the anchor, positive and negative of the i-th triplet, and by denoting as N the number of all possible triplets built in this way, we can write the loss in the following way:

$$L = \frac{1}{N} \sum_{i}^{N} \left[||g_w(x_a^{(i)}) - g_w(x_p^{(i)})||_2^2 - ||g_w(x_a^{(i)}) - g_w(x_n^{(i)})||_2^2 + \alpha \right]_+ , \tag{3}$$

where $\alpha \in \mathbb{R}$ denotes the margin. We optimize this loss with stochastic gradient descent over the parameters of the network, by drawing random triplets.

1.3 Experimental Details

SASL and TLSL were trained on the same 'pilot' simulated dataset containing 255 parameter and simulated data pairs. For both the SASL and TLSL the neural network is composed of 4 fully connected layers, with dimension of input neurons and outputs being equal to the dimension of data (9) and parameter (7) correspondingly, with hidden layers of size 14, 13 and 10, batch size 16 and with ReLU non-linearity. We trained the neural network for 1000 and 2000 epochs correspondingly for SASL and TLSL. Further the margin α for TLSL was chosen to be 1.

References

[1] P. Fearnhead and D. Prangle. Constructing summary statistics for approximate Bayesian computation: semi-automatic approximate Bayesian computation [with Discussion]. *Journal of the Royal Statistical Society. Series B (Statistical Methodology)*, 74(3):419–474, 2012. ISSN 1369-7412.

[2] B. Jiang, T.-y. Wu, C. Zheng, and W. H. Wong. Learning summary statistic for approximate bayesian computation via deep neural network. *Statistica Sinica*, pages 1595–1618, 2017.

[3] L. Pacchiardi, P. Künzli, M. Schöngens, B. Chopard, and R. Dutta. Distance-learning for approximate bayesian computation to model a volcanic eruption. *Sankhya B*, 83(1):288–317, 2021.

[4] F. Schroff, D. Kalenichenko, and J. Philbin. Facenet: A unified embedding for face recognition and clustering. In *Proceedings of the IEEE conference on computer vision and pattern recognition*, pages 815–823, 2015.
